# LTF regulates glioblastoma progression and temozolomide resistance via the NF-κB signaling pathway

**DOI:** 10.1590/1414-431X2025e13821

**Published:** 2025-09-12

**Authors:** Lili Gao, Hongbo Xiao, Hongyu Lu, Jun Ma, Haifeng Zhu

**Affiliations:** 1 Department of Medical Oncology, Taizhou City Hospital of Traditional Chinese and Western Medicine, Taizhou, Jiangsu, China; 2 Department of Neurosurgery, The People's Hospital of Rugao, Rugao, Jiangsu, China; 3 Department of Neurosurgery, The First Affiliated Hospital of Nanjing Medical University, Nanjing, Jiangsu, China; 4 Department of Neurosurgery, Nanjing First Hospital, Nanjing Medical University, Nanjing, Jiangsu, China; 5 Department of Neurosurgery, Taizhou City Hospital of Traditional Chinese and Western Medicine, Taizhou, Jiangsu, China

**Keywords:** Glioblastoma, Lactotransferrin, Nuclear factor-κB (NF-κB)

## Abstract

Glioblastoma (GBM) is the most prevalent tumor in the central nervous system in adults. Lactotransferrin (LTF) is a molecule involved in the growth of various tumors. However, the underlying mechanism of LTF in GBM progression and chemotherapy resistance remains unclear. In this study, the clinical and diagnostic value of LTF were evaluated. *In vitro* and *in vivo* experiments were performed to explore the functional role of LTF in GBM. Immunoprecipitation and immunofluorescence assays were performed to clarify the effect of LTF on nuclear factor-κB (NF-κB) activation. LTF was overexpressed in GBM and correlated with poor prognosis. LTF promoted GBM cell proliferation, invasion, and temozolomide (TMZ) resistance. Mechanism assay results indicated that LTF competitively binds to p65, rescuing the inhibited effect of PP2A on p65 phosphorylation, thereby activating the NF-κB signaling pathway. Our results confirmed that highly expressed LTF promoted GBM progression and TMZ resistance through the NF-κB signaling pathway.

## Introduction

Glioblastoma (GBM) is the most common malignant brain tumor and is highly resistant to temozolomide (TMZ) chemotherapy and radiation therapy (1,2). High proliferation, invasion, and resistance to therapies lead to poor prognosis of GBM patients, with less than 5% survival over five years (3,4). Revealing molecular mechanisms of GBM progression for the search of new therapeutic strategies is an urgent project to be carried out.

Lactotransferrin (LTF) is a well-known iron-binding protein, which exist in both tissues and secretions like breast milk (5-8). In addition to the iron delivery capacity, *LTF* can function as either an oncogene or a tumor suppressor (9,10). Since Bezault et al. (11) found that LTF inhibits the growth of solid tumors and development of experimental metastases in mice in 1994, LTF has been shown to exert anti-tumor activity for both prevention and treatment (12,13). However, LTF application in clinical trials is not satisfactory. Recently, more and more teams identified the oncological role of LTF. Wen et al. (14) reported that LTF induces radiation resistance in lung squamous cell carcinoma by promoting autophagy and forming an AMPK/SP2/NEAT1/miR-214-5p feedback loop. Qi et al. (15) demonstrated that down-regulated LTF enhances the radiosensitivity of nasopharyngeal carcinoma cells. In glioma, LTF was used as a coadjutor converting monodisperse iron oxide nanoparticles into glioma targeting agents (16). Arcella et al. (17) reported that exogenous LTF treatment inhibits GBM cell proliferation. However, the endogenous expression and function of LTF in GBM remains unknown.

In this study, we illustrate the expression and prognostic value of LTF in GBM using different datasets. Moreover, we explored the oncogenic activity of LTF by *in vitro* assays and *in vivo* experiments.

## Material and Methods

### Data acquisition

Transcriptional profiles of GBM and GTEx normal brain samples were searched from the Genomic Data Commons (GDC) at: https://portal.gdc.cancer.gov/projects/CPTAC-3. TCGA, CGGA, and Rembrandt data were collected from Gliovis (http://gliovis.bioinfo.cnio.es/). Bioinformatics analysis was performed using LinkedOmics (https://www.linkedomics.org/).

### Cell culture and transfection

Normal human astrocyte (NHA) cells and GBM cells (U87, D54, U251, LN18, and LN229) were purchased from the American Type Culture Collection (ATCC). GBM cells were cultured in Dulbecco's Modified Eagle's medium (DMEM) supplemented with 10% fetal bovine serum (FBS). NHA cells were cultured in astrocyte growth medium supplemented with insulin, rhEGF, GA-1000, L-glutamine, ascorbic acid, and 5% FBS. All cells were maintained in a humidified atmosphere at 37°C and 5% CO_2_. Polymerase chain reaction (PCR)-amplified human LTF and four phosphatases (PP1, PP2A, PP4, and WIP1) were cloned into pcDNA3.1 or pcDNA3.1/hygro (+)-Flag. Lentiviruses containing shRNA targeting LTF (5′-CCCUACAAACUGCGACCUGUA-3′) was synthesized from GeneChem (China). Lipofectamine 2000 Reagent was used for cell transfection according to the manufacturer's instructions (Beyotime Biotechnology, China).

### Western blot assay and antibodies

Protein isolation and western blot assays were performed as previously described (18). Antibodies against LTF (10933-1-AP), Cyclin E1 (11554-1-AP), Cyclin D1 (26939-1-AP), E-cadherin (20874-1-AP), N-cadherin (22018-1-AP), γ-H2AX (29380-1-AP), PP2A (13482-1-AP), GAPDH (10494-1-AP), and H3 (17168-1-AP) were from Proteintech (China). Antibodies against p65 (8242), p-p65 (3033), p-IKKβ (2078), p-IkKα (2859), and Flag (14793) were from Cell Signaling Technology (USA).

### Real-time quantitative PCR (RT-qPCR)

Total cellular RNA was extracted using TRIzol reagent as previously described (18). RNA was reverse transcribed into cDNA using PrimeScript RT Master Mix (Takara, China), and RT-qPCR was performed using Premix Ex TaqTM II (Takara). β-actin was used as an internal control (Table 1).

**Table 1:** Primers used in this study.

Name	Forward (5′ to 3′)	Reverse (5′ to 3′)
LTF	AGTCTACGGGACCGAAAGACA	CAGACCTTGCAGTTCGTTCAG
MCP-1	TCCCAAAGAAGCTGTGATCTTCA	TGCTTGTCCAGGTGGTCCAT
Bcl-xL	GAGGCAGGCGACGAGTTTGAA	GGGGTGGGAGGGTAGAGTGGA
CCL-20	TGATGTCAGTGCTGCTACTC	ATGTCACAGCCTTCATTGGC
A20	GCACACTGTGTTTCATCG	GGCATACATTGCTTGAAC
xIAP	ACGGATCTTTACTTTTGGGAC	CACCCTGGATACCATTTAGCAT
β-actin	CATGTACGTTGCTATCCAGGC	CTCCTTAATGTCACGCACGAT

RT-qPCR was performed using the SYBR Premix DimerEraser on a 7900HT system, and relative expressions were calculated by relative quantification (2^−△△Ct^).

### CCK8 assay

GBM transfected cells (1000/well) were seeded onto 96-well plates. Cells were incubated with CCK8 reagent (Dojindo Laboratories, Japan) for 2 h at 37°C. The absorbance was measured at 450 nm.

### Colony formation assay

Transfected GBM cells (800/well) were seeded onto 6-well plates. Two weeks later, cells were fixed and stained with crystal violet dye. ImageJ software (NIH, USA) was used for clone number counting.

### 5-ethynyl-2-deoxyuridine (EdU) assay

Transfected GBM cells (1000/well) were seeded onto 6-well plates for 24 h. The cell-light EdU imaging detection kit (Life Technologies, China) was used to stain cells according to the manufacturer's instructions. DAPI was used to stain nuclei. Finally, cells were photographed and the positive rate was calculated using ImageJ software.

### Transwell assay

A total of 1000 cells maintained in FBS-free DMEM were seeded onto a transwell chamber coated with extracellular interstitial gel. The chamber was placed in a 6-well plate, and DMEM with FBS was added onto the 6-well plate. Twenty-four hours later, cells that migrated to the lower membrane surface of the transwell chamber were fixed and stained with crystal violet. The migrated cells were imaged and counted using ImageJ software.

### *In vitro* 3D migration assay

GBM cells were transfected with green fluorescent protein and sorted by flow cytometry. A total of 1000 cells were resuspended with 10% Collagen I gels diluted by DMEM and seeded onto the ultra-low adsorption 96-well plates (Corning, USA). Twenty-four hours later, cells were photographed and the migratory zone was measured using ImageJ software.

### Flow cytometry assay

Flow cytometry assays were performed for apoptotic cell rate measurement. After TMZ treatment, cells were collected and stained with PI and Annexin V-FITC using the Apoptosis Detection Kit (Vazyme, China). Flow cytometry was used to detect apoptotic cells. The apoptotic cell rate was analyzed using FlowJo software.

### Immunofluorescent assay

A total of 500 GBM cells were seeded onto a glass-bottom confocal Petri dish. Twenty-four hours later, cells were fixed with 4% formaldehyde, blocked with 5% goat serum, and incubated with antibody against p65 at 4°C overnight. The next day, cells were washed and incubated with secondary antibodies. DAPI was used for nuclear staining. Fluorescence microscopy (Leica DMI3000B, Germany) was used for imaging.

### Immunoprecipitation assay

Cellular protein was isolated using a modified buffer (50 mM Tris-HCl [pH 7.5], 0.01% of SDS, 1% of Triton X-100, 150 mM NaCl, 1 mM dithiothreitol, 0.5 mM EDTA, 100 mM PMSF, 100 mM leupeptin, 1 mM aprotinin, 100 mM sodium orthovanadate, 100 mM sodium pyrophosphate, and 1 mM sodium fluoride). Anti-FLAG^®^ M2 magnetic beads were incubated with proteins overnight at 4°C with rotation. The next day, the complex was washed and prepared for western blot assay.

### Xenograft tumor assay

Four-week-old nude mice were purchased from GemPharmatech (China). The animal experiments were approved by Nanjing Medical University. To establish the intracranial tumor model, U251 cells were transfected with luciferase. 1×10^5^ U251-luciferase cells were implanted stereotactically into the brain of nude mice. Mice were treated or not with TMZ (66 mg/kg per day for 5 days). Tumor size was monitored by bioluminescence imaging at day 5 and day 30 (IVIS Spectrum; PerkinElmer, USA).

### Immunohistochemistry (IHC) assay

The mouse brains were collected, fixed with 4% formaldehyde, and embedded in paraffin. Tissue sections were stained with antibodies against LTF, Ki-67, MCP-1, and IgG as a negative control. The following proportion scores were assigned to the sections: 0 if 0% of tumor cells exhibited positive staining, 1 for >0 to 1% positive cells, 2 for 2 to 10% positive cells, 3 for 11 to 30% positive cells, 4 for 31 to 70% positive cells, and 5 for 71 to 100% positive cells. In addition, the staining intensity was scored on a scale of 0-3: 0, negative; 1, weak; 2, moderate; and 3, strong. The proportion and intensity scores were then added to obtain a total score ranging from 0 to 8.

### Statistical analysis

All data are reported as means±SE, and statistical analyses were performed with GraphPad software. Student's *t*-test, chi-squared test, or one-way analysis of variance (ANOVA) was used compare differences between groups. The Kaplan-Meier method with the log-rank test was used to calculate the overall survival (OS) rate for comparison between different groups. P<0.05 was considered as statistically significant.

## Results

### LTF was upregulated in GBM and correlated with poor prognosis

**Figure 1:**
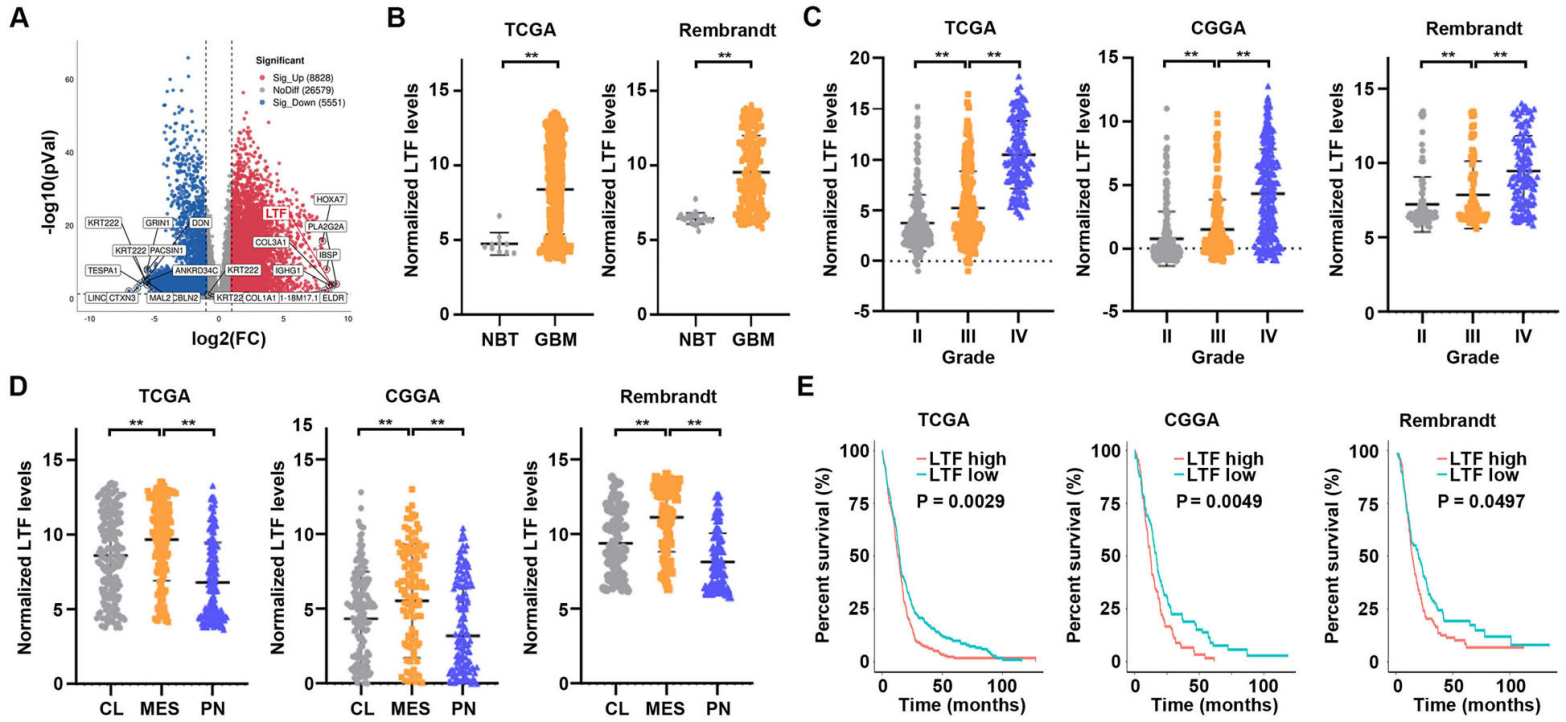
Lactotransferrin (LTF) is upregulated in glioblastoma (GBM) and correlated with poor prognosis. **A**, Volcano plot of differentially expressed mRNAs in GBM compared with normal brain samples. **B**, LTF expression levels in normal brain tissues (NBT) and GBM samples according to TCGA and Rembrandt datasets. **C**, LTF expression levels in different grades of glioma samples according to TCGA, CGGA, and Rembrandt datasets. **D**, LTF expression levels in mesenchymal (MES), proneural (PN), and classical (CL) samples according to TCGA, CGGA, and Rembrandt datasets. **E**, Kaplan-Meier analysis of overall survival of GBM samples according to LTF expression using TCGA, CGGA, and Rembrandt datasets. **P<0.01, ANOVA.

To explore potential therapeutic targets in GBM, we employed the transcriptional analysis results of 99 GBMs and 10 unmatched GTEx normal brain samples from Wang et al. (19). With the screening strategy (log_2_FC ≥1, P value <0.05), we selected LTF, the mostly upregulated gene in GBM samples, as the research target (Figure 1A). The data from TCGA and Rembrandt datasets also revealed that LTF was highly expressed in GBM compared to normal brain tissues (Figure 1B). In addition, we found that LTF expression levels were significantly increased in high grade glioma samples compared to low grade samples using TCGA, CGGA, and Rembrandt datasets (Figure 1C). We further analyzed the distribution of LTF expression in different molecular subtypes of glioma using TCGA, CGGA, and Rembrandt datasets. As a result, LTF was dramatically increased in mesenchymal compared to proneural and classical samples, indicating the tumor-promoting function of LTF in glioma (Figure 1D). Finally, we analyzed the potential role of LTF in predicting patient prognosis. Using TCGA, CGGA, and Rembrandt datasets, we found that increased LTF expression was associated with poor overall survival of GBM patients (Figure 1E). In summary, LTF can be considered a potential biomarker for GBM.

### Silencing LTF inhibited GBM cell proliferation and invasion

**Figure 2:**
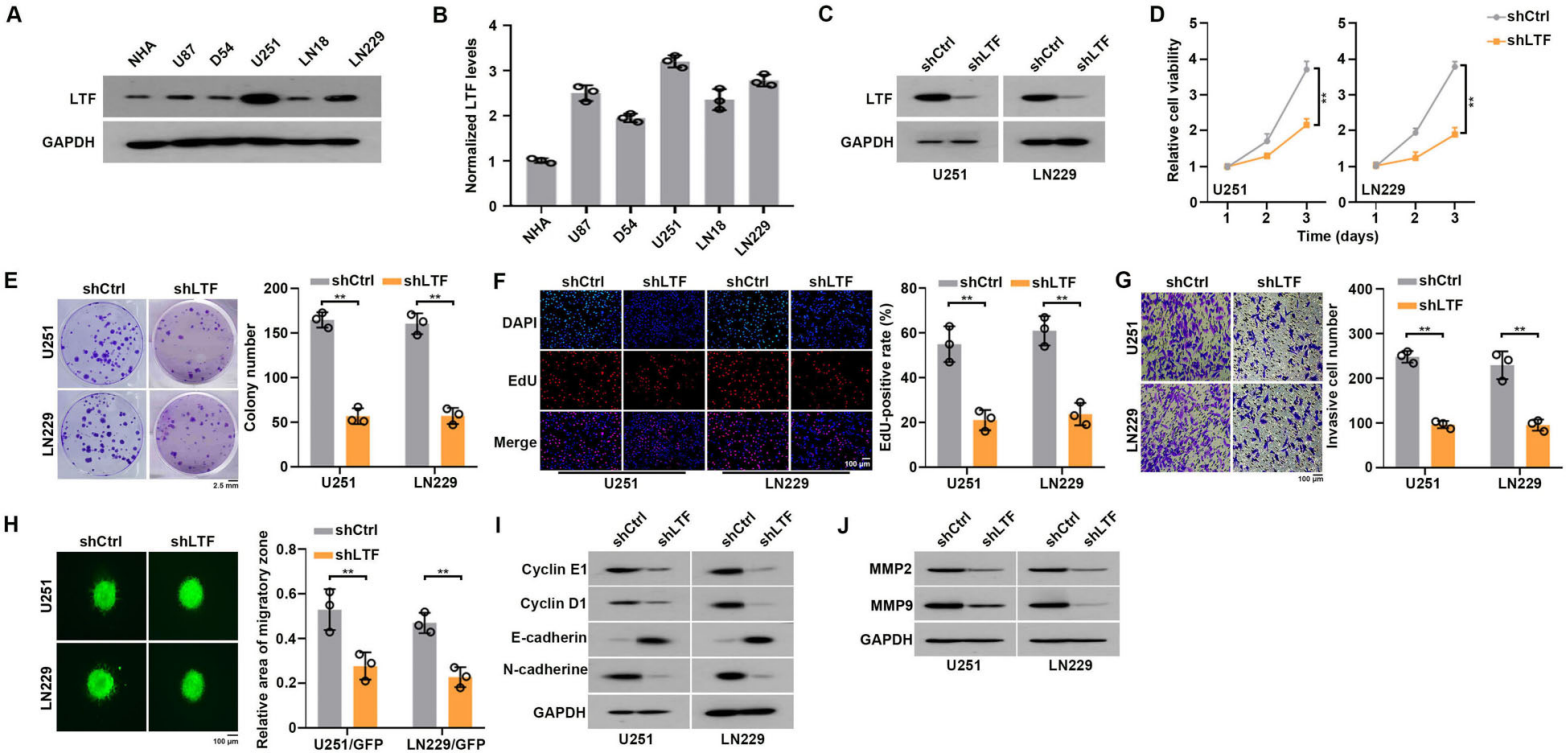
Silencing lactotransferrin (shLTF) inhibited glioblastoma (GBM) cell proliferation and invasion. **A**, LTF expression levels in normal human astrocyte (NHA) and GBM cells were measured by western blot. **B**, LTF expression levels in NHA and GBM cells were measured by RT-qPCR. **C**, Knockdown efficiency of LTF in GBM cells was confirmed by western blot. **D**, Cell growth of control and LTF-depleted GBM cells was measured by CCK8 assays. **E**, Cell growth of control and LTF-depleted GBM cells was measured by colony formation assays and **F**, EdU assays. **G**, Cell invasion of control and LTF-depleted GBM cells was measured by transwell assays and **H**, *in vitro* 3D migration assays. **I**, Proliferation and EMT associated protein levels in control and LTF-depleted GBM cells were measured by western blot. **J**, Invasion associated protein levels in control and LTF-depleted GBM cells were measured by western blot. Data are reported as means and SE. **P<0.01, Student's *t*-test. Scale bar 100 μm.

Firstly, we detected protein and mRNA levels of LTF in NHA and GBM cells. As LTF was highly expressed in U251 and LN229 cells (Figure 2A and B), we chose the two cell lines as research models. To explore the function of LTF in GBM cells, we synthesized control or shRNA-targeting LTF and successfully transfected them into U251 and LN229 cells (Figure 2C). As shown in Figure 2D, LTF knockdown significantly suppressed GBM cell growth. Results of colony formation and EdU assays also indicated that LTF knockdown significantly suppressed GBM cell proliferation (Figure 2E and F). Furthermore, we evaluated whether LTF is involved in GBM cell invasion. Transwell assays demonstrated that slicing LTF dramatically impaired the invasive capacity of U251 and LN229 cells (Figure 2G). In addition, we transfected U251 and LN229 cells with GFP and performed *in vitro* 3D migration assays. The results indicated that LTF knockdown inhibited the invasive zone of GBM cells (Figure 2H). Finally, we determined the expression of the proliferation- and invasion-associated molecules in control or LTF depleted cells. Cyclin E1, Cyclin D1, and N-cadherine protein levels were markedly decreased while E-cadherin protein levels were dramatically increased upon LTF knockdown (Figure 2I). To further investigate the mechanism whereby LTF promoted GBM cell invasion, we determined the expression of the invasion-associated molecules, matrixmetalloproteinase (MMP)-2 and MMP-9. The results indicated that LTF deletion decreased MMP-2 and MMP-9 expression (Figure 2J). In summary, LTF depletion suppressed GBM cell proliferation and invasion.

### LTF overexpression promoted GBM cell proliferation and invasion

**Figure 3:**
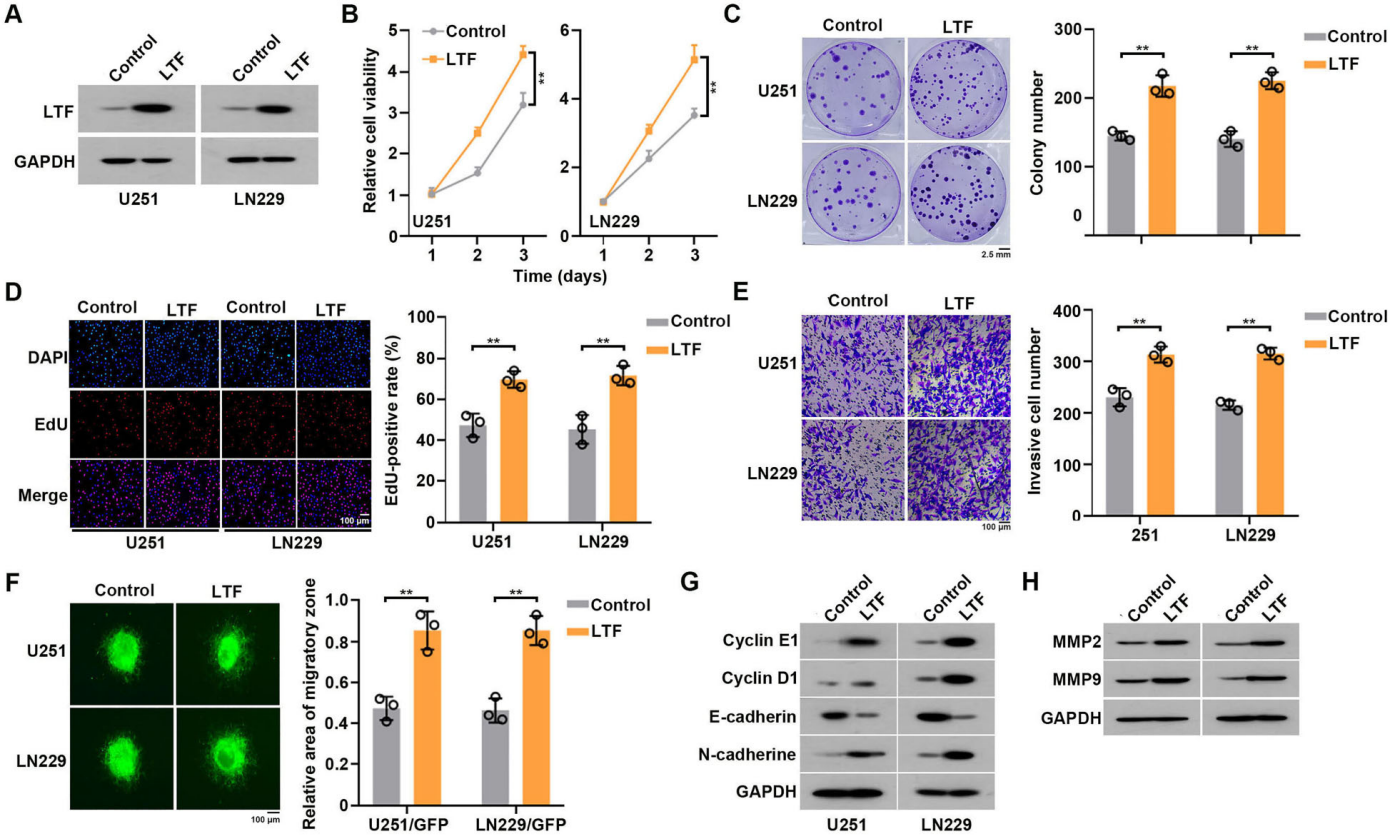
Lactotransferrin (LTF) overexpression promoted glioblastoma (GBM) cell proliferation and invasion. **A**, Overexpression efficiency of LTF in GBM cells was confirmed by western blot. **B**, Cell growth of control and LTF overexpressed GBM cells was measured by CCK8 assays. **C**, Cell growth of control and LTF overexpressed GBM cells was measured by colony formation assays. Scale bar 2.5 mm. **D**, Cell growth of control and LTF overexpressed GBM cells was measured by EdU assays. **E**, Cell invasion of control and LTF overexpressed GBM cells was measured by transwell assays. **F**, Cell invasion of control and LTF overexpressed GBM cells was measured by *in vitro* 3D migration assay. **G**, Proliferation and epithelial-to-mesenchymal transition (EMT)-associated protein levels in control and LTF overexpressed GBM cells were measured by western blot. **H**, Invasion-associated protein levels in control and LTF overexpressed GBM cells was measured by western blot. Data are reported as means and SE. **P<0.01, Student's *t*-test. Panels D, E, and F: scale bar 100 μm.

To further assess the biological function of LTF in GBM cells, we overexpressed LTF in U251 and LNN229 cells (Figure 3A). Results of CCK8, colony formation, and EdU assays suggested that LTF overexpression significantly promoted cell growth and proliferation capacity (Figure 3B-D). As expected, LTF overexpression also promoted GBM cell invasion (Figure 3E and F). Western blot results showed that LTF overexpression led to a significant increase of Cyclin E1, Cyclin D1, N-cadherin and decrease of E-cadherin protein levels (Figure 3G). In addition, LTF overexpression increased MMP-2 and MMP-9 expression (Figure 3H). In summary, LTF overexpression promoted proliferation and invasion of GBM cells.

### LTF promoted chemoresistance of GBM cell

**Figure 4:**
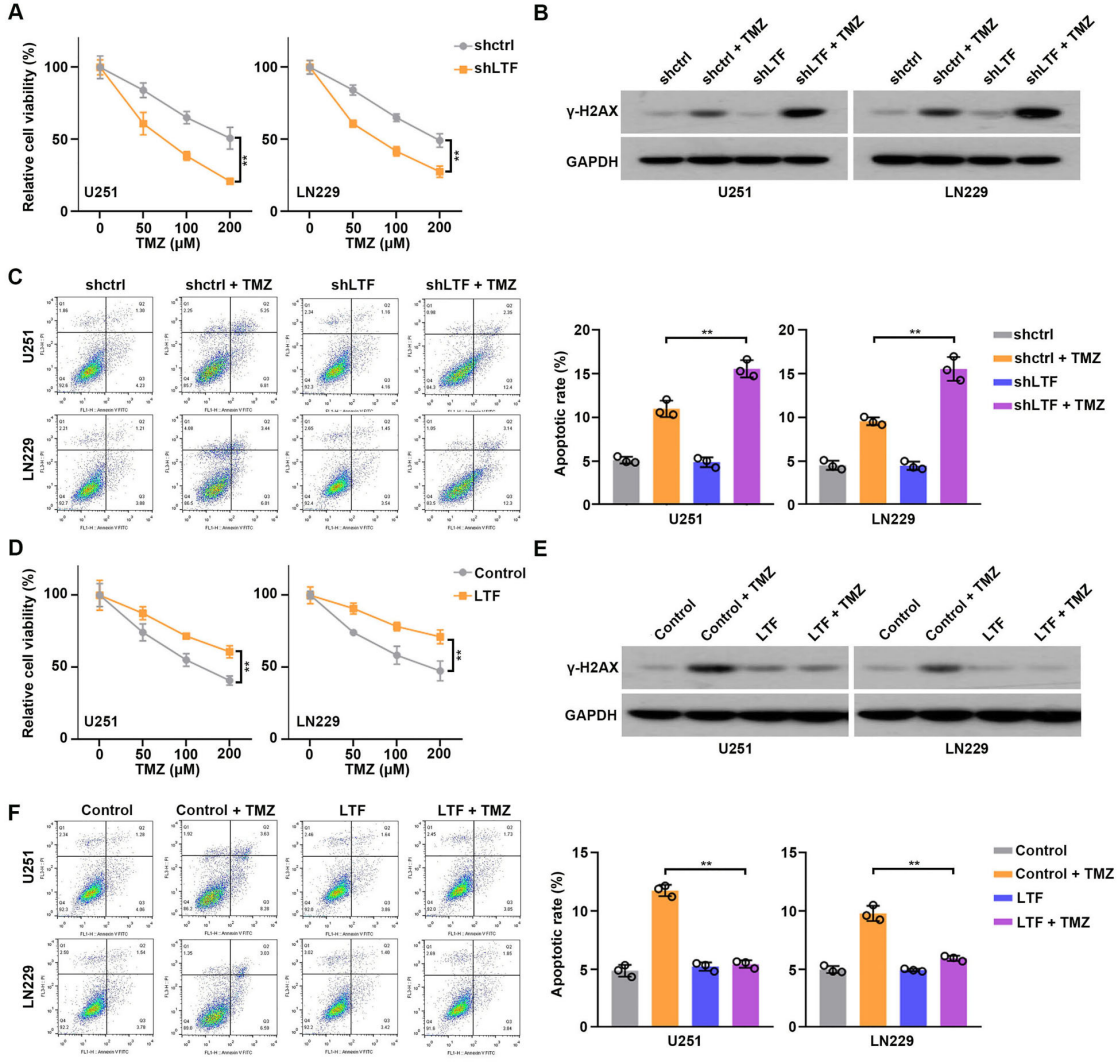
Lactotransferrin (LTF) promoted chemoresistance of glioblastoma (GBM) cells. **A**, Control and LTF-depleted GBM cells were treated with increasing doses of TMZ for 24 h, and cell growth was measured by CCK8 assays. **B**, Control and LTF-depleted GBM cells were treated with or without TMZ for 24 h, and γ-H2AX expression was measured by western blot. **C**, Control and LTF-depleted GBM cells were treated with or without TMZ for 24 h, and apoptotic rate was measured by flow cytometry. **D**, Control and LTF-overexpressed GBM cells were treated with increasing doses of TMZ for 24 h, and cell growth was measured by CCK8 assays. **E**, Control and LTF-overexpressed GBM cells were treated with or without TMZ for 24 h, and γ-H2AX expression was measured by western blot. **F**, Control and LTF-overexpressed GBM cells were treated with or without TMZ for 24 h, and apoptotic rate was measured by flow cytometry. Data are reported as means and SE. **P<0.01, ANOVA.

Next, we investigated the effect of LTF on TMZ resistance in GBM cells. We treated control or LTF-depleted U251 and LN229 cells with an increased dose of TMZ, and CCK8 results indicated that LTF knockdown significantly increased the sensitivity of GBM cells to TMZ (Figure 4A). We further detected the apoptotic cell rate of GBM cells, and the results of western blot and flow cytometry assays indicated that LTF knockdown dramatically increased apoptosis of GBM cells after TMZ treatment (Figure 4B and C). At the same time, we estimated the response of GBM cells to TMZ after LTF overexpression. CCK8 results demonstrated significantly decreased TMZ sensitivity of LTF-overexpressed cells (Figure 4D). Meanwhile, LTF overexpression decreased apoptosis of GBM cells after TMZ treatment (Figure 4E and F). These results suggested that LTF was involved in TMZ resistance.

### LTF regulated NF-κB signaling activation

**Figure 5:**
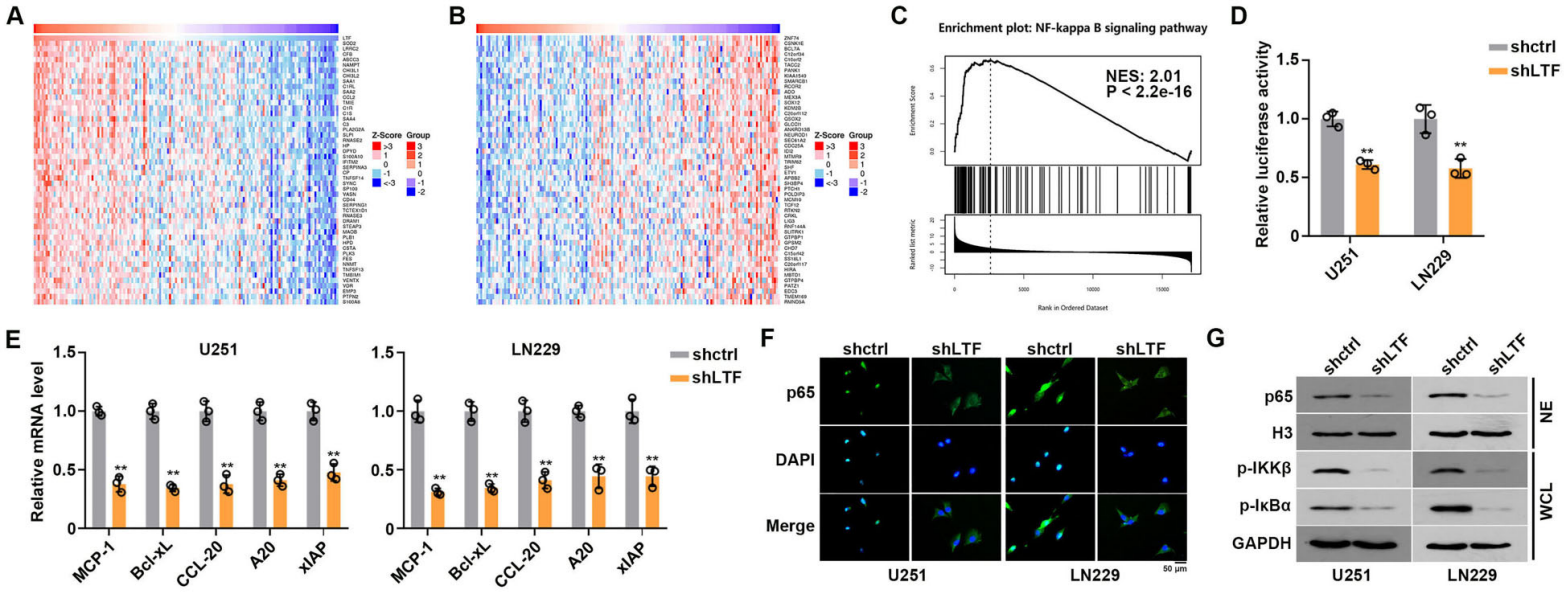
Lactotransferrin (LTF) regulated nuclear factor (NF)-κB signaling activation. **A**, Top 50 positive coexpression genes of LTF in glioblastoma (GBM) analyzed using TCGA dataset. **B**, Top 50 negative coexpression genes of LTF in GBM analyzed using TCGA dataset. **C**, GSEA analysis indicated that NF-κB signaling is associated with high LTF expression. **D**, Luciferase assay of the interaction between NF-κB signaling and LTF in GBM cells. **E**, mRNA expression levels of NF-κB target genes was measured by RT-qPCR. **F**, p65 distribution in control and LTF-depleted GBM cells were analyzed by immunofluorescence assays. Scale bar 50 μm. **G**, p65 distribution and phosphorylation levels of IκBα and IKKβ in control or LTF-depleted GBM cells were analyzed by western blot. Data are reported as means and SE. **P<0.01, ANOVA.

To identify the mechanism of LTF in glioma, we applied bioinformatics analysis of LTF related molecules by LinkedOmics tool. The positive and negative molecules of LTF in GBM were presented (Figure 5A and B). GSEA was performed and the NF-κB signaling pathway was found to be positively regulated by LTF (Figure 5C). According to bioinformation analysis results, we investigated whether LTF regulates NF-κB signaling pathway activation. Luciferase assay results indicated that LTF knockdown significantly suppressed NF-κB signaling pathway activation (Figure 5D). Moreover, we measured the mRNA expression levels of NF-κB target genes (*MCP-1*, *Bcl-xL*, *CCL-20*, *A20*, *XIAP*). The results suggested that LTF knockdown dramatically inhibited mRNA levels of these genes (Figure 5E). In addition, immunofluorescence and western blot results showed a decrease of nuclear p65 accumulation after LTF knockdown (Figure 5F and G). We also assessed the phosphorylation levels of IkBα and IKKβ. Western blot results indicated that LTF knockdown significantly decreased the phosphorylation levels of IkBα and IKKβ (Figure 5G).

### LTF competitively bound p65

**Figure 6:**
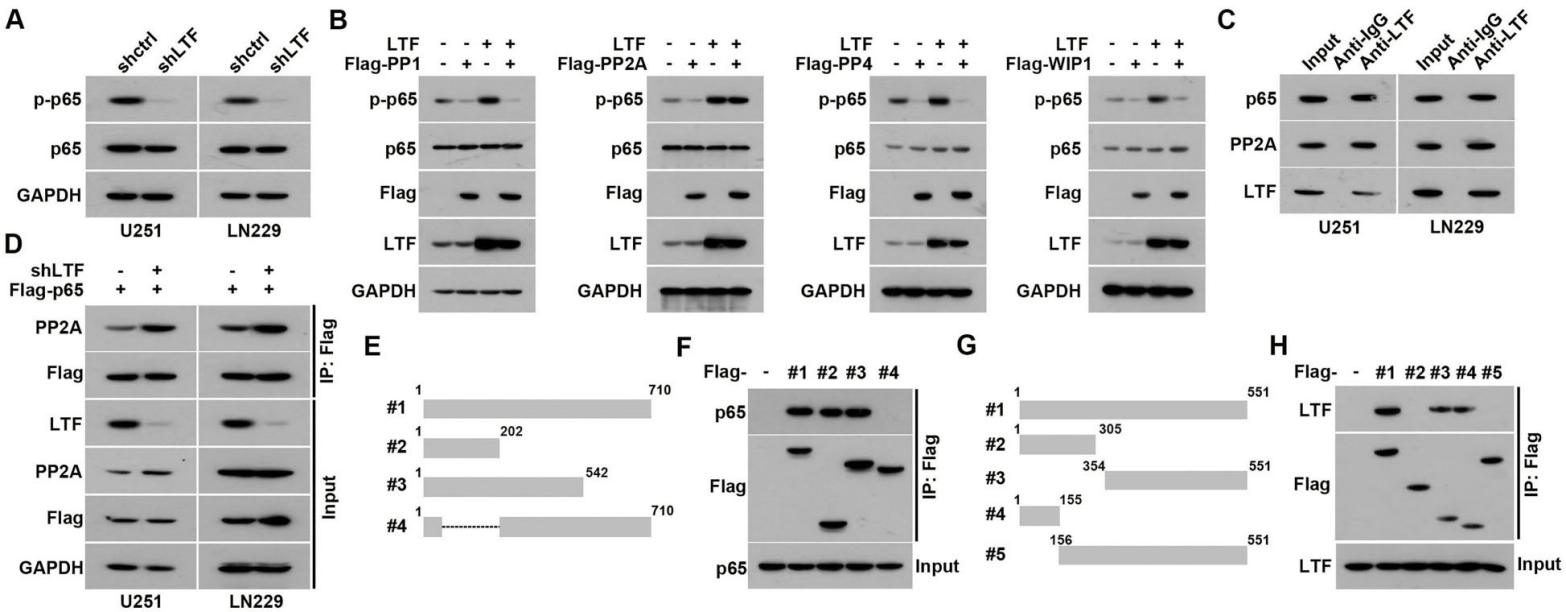
Lactotransferrin (LTF) competitively bound p65. **A**, Phosphorylation levels of p65 in control or LTF-depleted glioblastoma (GBM) cells was analyzed by western blot. **B**, U251 cells were transfected with LTF with or without four phosphatases. Phosphorylation levels of p65 in control or LTF-depleted GBM cells were analyzed by western blot. **C**, Immunoprecipitates were prepared with anti-LTF antibody. p65 and PP2A expression in immunoprecipitates was measured by western blot. **D**, Control and LTF-depleted cells were transfected with Flag-p65. Immunoprecipitates were prepared with anti-Flag antibody. PP2A expression in immunoprecipitates was measured by western blot. **E**, Schematic representation of Flag-tagged full-length LTF (#1) and various deletion mutants (#2-4). **F**, Immunoprecipitation and western blot assays using Flag antibody showing the interaction between p65 and LTF protein in U251 cells transfected with a series of truncations of Flag-tagged LTF. **G**, Schematic representation of Flag-tagged full-length p65 (#1) and various deletion mutants (#2-5). **H**, Immunoprecipitation and western blot assays using Flag antibody showing the interaction between p65 and LTF protein in U251 cells transfected with a series truncations of Flag-tagged p65.

To further probe the regulation of LTF in p65 nuclear transfer, we examined the phosphorylation levels of p65 in control or LTF-depleted cells. The results indicated that LTF knockdown markedly restrained the phosphorylation levels of p65 (Figure 6A). Previous studies found that four phosphatases (PP1, PP2A, PP4, and WIP1) negatively regulate p65 phosphorylation via dephosphorylation (20). As shown in Figure 6B, the overexpression of the four phosphatases significantly decreased phosphorylation levels of p65 in U251 cells (Figure 6B). Moreover, we co-transfected LTF in cells. The western blot results indicated that LTF only restored the p65 phosphorylation levels suppressed by PP2A (Figure 6B). Co-immunoprecipitation results indicated that LTF could interact with both p65 and PP2A in GBM cells (Figure 6C). Further co-immunoprecipitation results showed that LTF knockdown promoted interaction between p65 and PP2A (Figure 6D). These results raised the possibility that LTF may disrupt the dephosphorylation effect of PP2A on p65. To verify this hypothesis, we constructed truncated mutation of LTF and p65 (Figure 6E and G). Co-immunoprecipitation results indicated that the N-terminal of LTF and partial C-terminal (1-155) of p65 were responsible for the interaction between LTF and p65 (Figure 6F and H). Coincidentally, PP2A negatively regulated p65 phosphorylation by binding with 1-155 domain of p65 (21). Therefore, LTF competitively bound to p65, which ameliorated the inhibition of PP2A on p65 phosphorylation.

### LTF promoted GBM progression by activating the NF-κB signaling pathway

**Figure 7:**
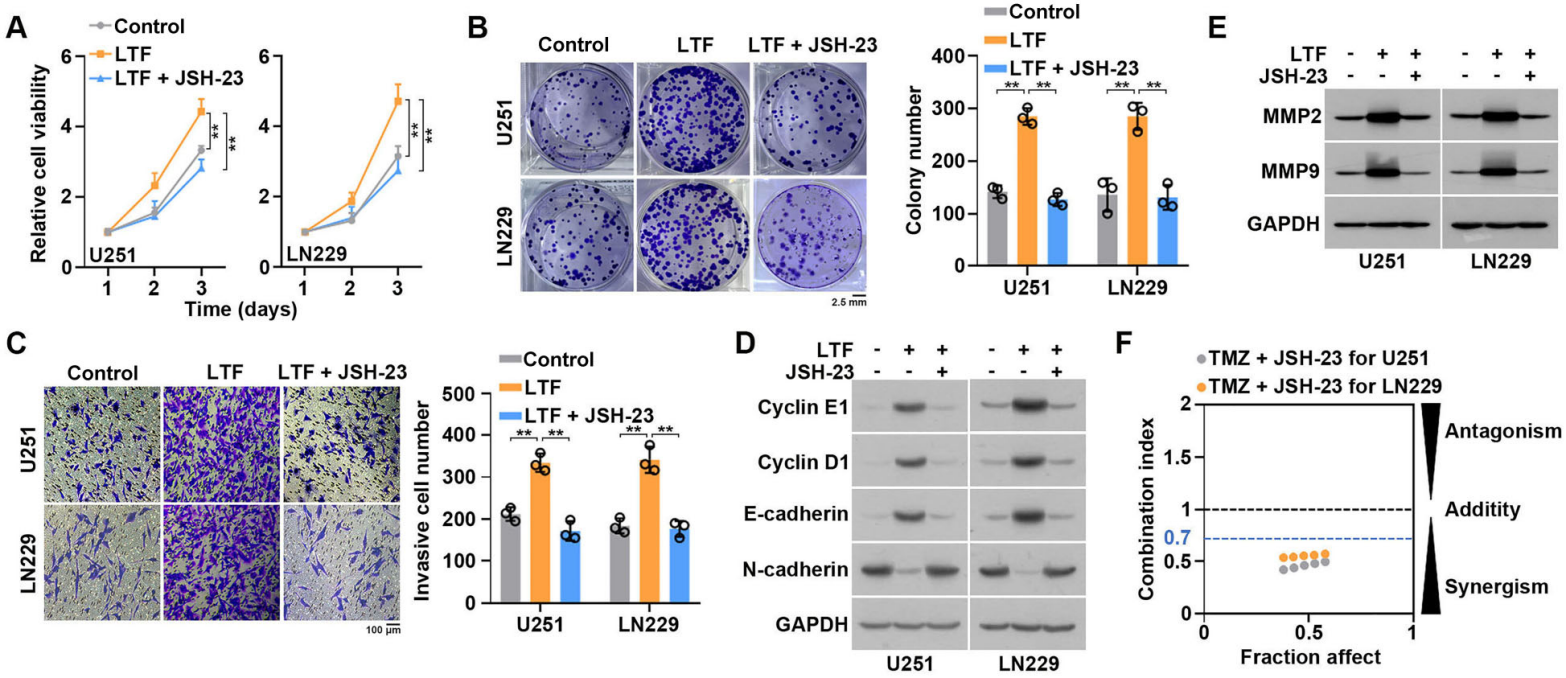
Lactotransferrin (LTF) promoted glioblastoma (GBM) progression by activating the NF-κB signaling pathway. **A**, LTF-overexpressed GBM cells were treated with JSH-23 for 24 h, and cell growth was measured by CCK8 assays. **B**, LTF-overexpressed GBM cells were treated with JSH-23 for 24 h, and cell growth was measured by colony formation assays. Scale bar 2.5 mm; **C**, LTF-overexpressed GBM cells were treated with JSH-23 for 24 h, and cell invasion was measured by transwell assays. Scale bar 100 μm. **D**, LTF-overexpressed GBM cells were treated with JSH-23 for 24 h, and proliferation and EMT associated protein levels were measured by western blot. **E**, LTF-overexpressed GBM cells were treated with JSH-23 for 24 h, and invasion-associated protein levels were measured by western blot. **F**, Control and LTF-overexpressed GBM cells were treated with increasing doses of TMZ and JSH-23. The combination index (CI) and the fraction affected by the dose (Fa) for the combination of JSH-23 and TMZ were calculated using CalcuSyn software (version 2; Biosoft). 0<CI<1 indicates a synergistic interaction. Data are reported as means and SE. **P<0.01, ANOVA.

Next, we investigated whether the NF-κB signaling pathway was involved in LTF-mediated malignant biological behavior. JSH-23 is an NF-κB inhibitor, which can inhibit nuclear transfer of p65. JSH-23 was used to treat control or LTF-overexpressed GBM cells. CCK8 and colony formation assays demonstrated that JSH-23 abolished LTF-mediated promotion of cell growth (Figure 7A and B). Moreover, transwell assays indicated that JSH-23 partially abolished LTF-mediated promotion of cell invasion (Figure 7C). Western blot results indicated that JSH-23 treatment reversed the expression of cell proliferation and invasion biomarkers (Figure 7D and E). Moreover, JSH-23 exhibited a strongly synergistic effect with TMZ for GBM cell treatment (Figure 7F). Taken together, LTF promoted GBM malignant phenotypes by activating the NF-κB signaling pathway.

### LTF depletion inhibited tumor growth and sensitized tumor to TMZ *in vivo*

**Figure 8:**
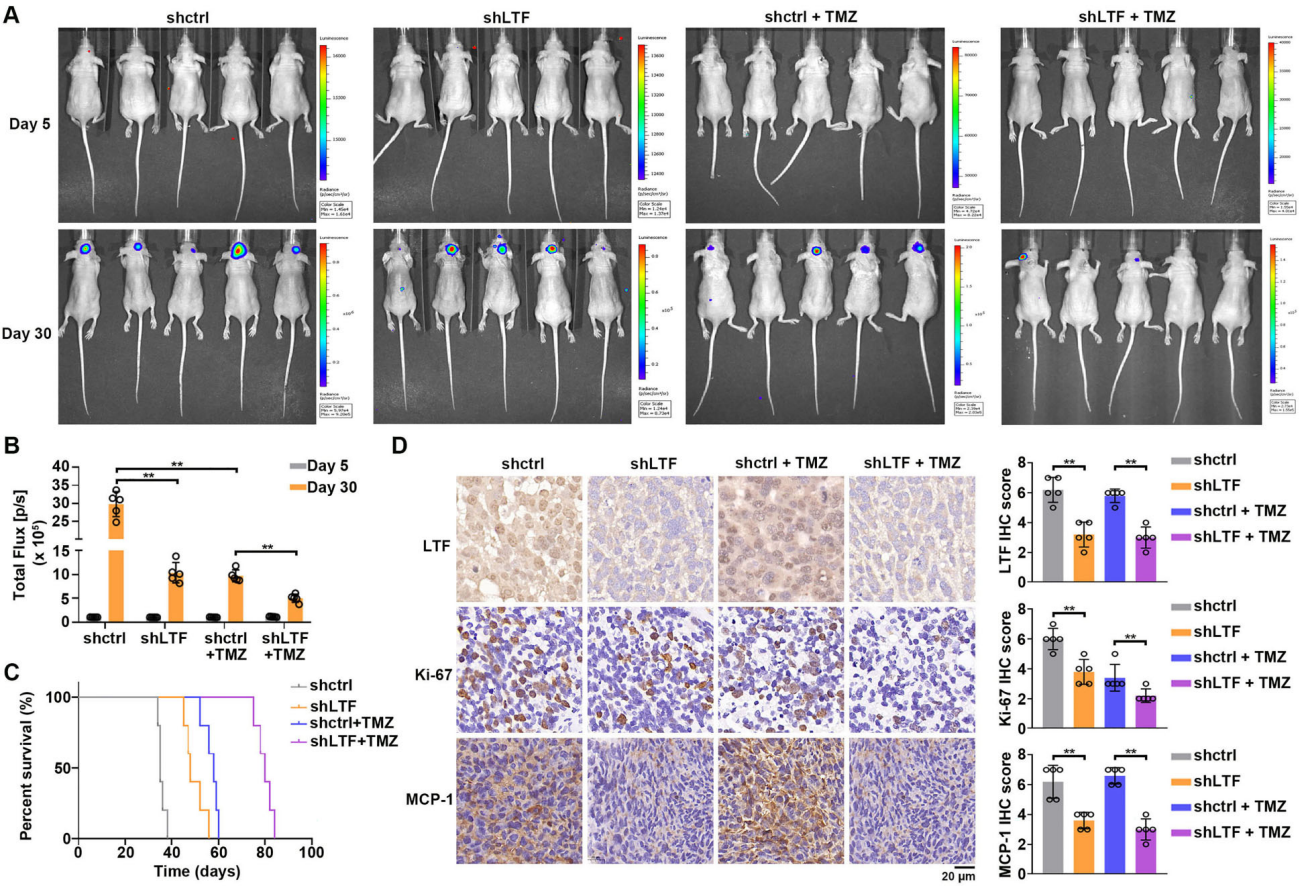
Lactotransferrin (LTF) depletion inhibited tumor growth and sensitized the tumor to TMZ *in vivo.* **A**, Bioluminescent images of intracranial glioblastoma (GBM) xenografts derived from control or LTF-depleted U251 cells. **B**, Quantification of bioluminescent images. **C**, Kaplan-Meier survival curves of intracranially injected mice. **D**, LTF, Ki67, and MCP-1 levels in tumors were measured by IHC assays. Scale bar 20 μm. Quantification of IHC is shown on the right. Data are reported as means and SE. **P<0.01, ANOVA.

The functional role of LTF on promoting GBM progression and TMZ resistance was evaluated *in vivo* using the orthotopic GBM model. Control and LTF-depleted U251 cells were transfected with luciferase and injected into the brains of mice. Seven days after implantation, mice were treated with placebo or TMZ for 5 days; a total of 2 cycle treatments were performed. Representative bioluminescence images of mice are shown in Figure 8A. Statistical analysis suggested that LTF depletion significantly suppressed tumor growth similar to the effect of TMZ treatment (Figure 8B). Compared with control tumors, LTF knockdown tumors were more sensitive to TMZ treatment (Figure 8B). The median survival of mice bearing LTF-depleted cells was significantly longer compared with control mice (Figure 8C). Moreover, TMZ treatment contributed to longer survival time of mice bearing LTF-depleted tumors than mice bearing control tumors (Figure 8C). IHC results showed that decreased LTF levels were accompanied by decreased Ki-67 and MCP-1 levels in tumors (Figure 8D). Taken together, LTF promoted GBM progression and TMZ resistance by activating the NF-κB signaling pathway *in vivo*.

## Discussion

In this study, RNA expression data from bulk GBM samples indicated that LTF is one of the most upregulated molecules in GBM. Public datasets also indicate that LTF expression is associated with glioma grade and molecular subtypes. High LTF is a potential biomarker for poor prognosis. Moreover, we demonstrated that LTF is an oncogenic driver in GBM proliferation, invasion, and TMZ resistance by activating the NF-κB signaling pathway.

LTF, known as a “miracle molecule”, is a component of the whey protein that is abundantly present in mammal milk (22). In addition, LTF has also been detected in many organs and cells of the human body (23). Structural analysis showed that LTF consists of a carboxyl terminal (C terminal), an amino terminal (N terminal), and an α helix (24). The N-linked glycosylation plays a vital role in LTF stability sustention (25). The “miracle” character of LTF is due to its multifaceted effects. In addition to binding iron, LTF is able to modulate lipid metabolism, decrease oxidative stress, and regulate tumor progression (26). Traditional knowledge indicated the anticancer role of LTF in various kinds of tumors. However, recent studies demonstrated the potential oncogenic role of LTF. LTF was reported to be involved in resistance to radiation therapy in lung squamous cell carcinoma and nasopharyngeal carcinoma (14,15). In glioma, Arcella et al. (17) used LTF to treat GBM cells and found that external LTF transitorily downregulated Cyclin D1 and Cyclin D4 expression in glioma cells. Our results showed a significant increase of Cyclin D1 and Cyclin E1 in U251 and LN229 cells 48 h after transfection. Moreover, we depleted LTF in GBM cells, and cellular and animal results indicated that loss of LTF significantly inhibited tumor progression. Therefore, we considered that *LTF* functions as an oncogene in GBM.

Nuclear factor-κB (NF-κB) is a heterodimeric complex consisting of two members of the following five molecules: p50, p52, Rel-like domain containing protein A (RelA/p65), RelB/RELB (RelB), and c-Rel/REL (c-Rel). Without stimulations by TNFα, NF-κB dimers activation is suppressed by IκBα binding and retarded in cytoplasm. Upon reception of the tumor necrosis factor (TNF)-α stimulation, IκBα is phosphorylated by IKKβ followed by ubiquitination and proteosomal degradation, which dissociate NF-κB dimers to the nucleus (27,28). NF-κB signaling activation has been found in GBM tissues compared with non-GBM tissues (29,30). Previous studies demonstrated that NF-κB signaling activation contributes to TMZ resistance in glioma. Huang et al. (31) performed a genome-wide CRISPR-Cas9 screening in glioma cells and found that NF-κB/E2F6 axis is responsible for EGFRvIII-associated TMZ resistance. Inhibition of nuclear factor kappa-B subunit epsilon (IKBKE), an activator of the NF-κB signaling pathway, increases TMZ resistance by upregulating MGMT activity (32). However, the precise mechanism of NF-κB activation in GBM remains largely undefined. In addition, the clinical trial using sulfasalazine, an NF-κB inhibitor, on recurrent anaplastic astrocytoma or GBM patients was terminated owing to a lack of response (33). Hence, more studies about NF-κB in GBM are needed to improve the response. Our results indicated that LTF could significantly activate NF-κB signaling by promoting nuclear p65. Interestingly, LTF could directly interact with p65. In addition, for the first time we found that LTF could increase p65 phosphorylation level by competitively inhibiting the binding of PP2A with p65.

Taken together, the findings of this study demonstrated that LTF acted as an oncogenic regulator in GBM by promoting NF-κB signaling pathway activation, extending knowledge on the molecular mechanism of GBM progression and providing new potential therapeutic targets for GBM treatment.
